# Assessing the Use of Microlearning for Preceptor Development

**DOI:** 10.3390/pharmacy11030102

**Published:** 2023-06-15

**Authors:** Stephanie M. Roskowski, Michael D. Wolcott, Adam M. Persky, Denise H. Rhoney, Charlene R. Williams

**Affiliations:** 1 Eshelman School of Pharmacy, University of North Carolina Chapel Hill, 301 Pharmacy Lane, CB #7574, Chapel Hill, NC 27599, USA; 2 HPU Workman School of Dental Medicine, One University Parkway, High Point, NC 27268, USA; 3 Eshelman School of Pharmacy, University of North Carolina Chapel Hill, 220 Campus Drive, Asheville, NC 28804, USA

**Keywords:** microlearning, preceptor development, pharmacy, technology, learning, teaching, efficiency, education, engagement

## Abstract

The objective of this study was to evaluate microlearning as a preceptor development method compared to a traditional method of learning. Twenty-five preceptor participants volunteered to engage in a learning intervention about two preceptor development topics. Participants were randomized 1:1 to either a thirty-minute traditional learning experience or a fifteen-minute microlearning experience; participants then crossed over to the other intervention for comparison. Primary outcomes were satisfaction, changes in knowledge, self-efficacy, and perception of behavior, confidence scale, and self-reported frequency of behavior, respectively. One-way repeated measures ANOVA and Wilcoxon paired t-tests were used to analyze knowledge and self-efficacy, and Wilcoxon paired t-tests were utilized to assess satisfaction and perception of behavior. Most participants preferred microlearning over the traditional method (72% vs. 20%, *p* = 0.007). Free text satisfaction responses were analyzed using inductive coding and thematic analysis. Participants reported that microlearning was more engaging and efficient. There were no significant differences in knowledge, self-efficacy, or perception of behavior between microlearning and the traditional method. Knowledge and self-efficacy scores for each modality increased compared to the baseline. Microlearning shows promise for educating pharmacy preceptors. Further study is needed to confirm the findings and determine optimal delivery approaches.

## 1. Introduction

Optimization of preceptor development programs is essential for the efficient and effective education of pharmacists who serve as mentors to students during their pharmacy practice experiences. Preceptors commonly have many time constraints in a practice environment [1,2,3,4,5]. It has become increasingly difficult to educate pharmacy preceptors in a quick and effective manner as there is often not enough time for them to attend preceptor development programming as well as to fully implement information from these programs [6]. Short delivery times have been requested for medical faculty development programming [7]. Additionally, engaging, effective, and efficient delivery of pharmacy preceptor development content is suggested [8].

Microlearning is a strategy of acquiring knowledge or skills in small units, which can be as short as a few seconds to 15 min as a way to facilitate training and continuing education. By breaking down broad concepts into smaller components, learners are able to attain several educational benefits that may be less prominent in traditional methods, such as increased engagement, knowledge retention, and a higher capacity to transfer and apply new knowledge to practice [9,10].

The majority of microlearning studies in health professions education settings evaluate its use in classroom or experiential settings for students [11]. The results show that microlearning can improve student knowledge as evidenced by higher class grades or improved student performance [9,12]. Microlearning has not been extensively evaluated as a means to further improve pharmacy preceptor development, and microlearning learning outcomes for preceptors are lacking at this time [8,13,14]. In medical education, the “snippet” faculty development approach has been used to train educators using a hands-on 15–20 min workshop with multiple teaching modalities [15]. Satisfaction was positive, but learning outcomes were not reported [15]. Dyrbe and colleagues noted that five-minute multimedia videos developed for faculty at academic health centers had high open rates and satisfaction [16]. More information is needed on the effect of microlearning on preceptor knowledge, self-efficacy, and behavior as well as pharmacy preceptor satisfaction.

Introducing microlearning into the pharmacy preceptor development space may offer a solution to training with time constraints given its reduced delivery time, yet more information on outcomes is needed to support its use. The objectives of this study were to assess microlearning as a preceptor development modality by evaluating satisfaction, change in preceptor knowledge, self-efficacy, and perceived behavior as compared to a longer traditional learning technique.

## 2. Materials and Methods

This was a prospective randomized, crossover-controlled study conducted at the University of North Carolina Eshelman School of Pharmacy. Participants were recruited by convenience sampling via email from a pool of 531 active pharmacy preceptors who had not been recently requested to participate in other research projects. Convenience sampling was utilized due to participant availability and willingness to participate in the study. Potential participants received an email with the study details, and informed consent was obtained from all subjects involved in the study.

Inclusion criteria consisted of preceptors from the UNC Eshelman School of Pharmacy who had been precepting for at least one year. Preceptors who had been practicing for less than a year, those that indicated a strong knowledge base of any of the topics included in the study, and those preceptors who had recently been invited to participate in other research were excluded from the study.

Participants engaged in a learning intervention about two precepting methods, each presented using a different modality (i.e., traditional modality and microlearning). The first method focused on the three-step Pause, Predict, Ponder method, which teaches preceptors how to prompt learners to make a prediction in order to improve their memory and clinical reasoning skills. Student learners are prompted by the preceptor to first pause at a key moment, make a prediction on a key concept based on their previous knowledge, and then reflect on the solution and how it compared to their prediction [17]. The second method was a novel five-step strategy termed LEARNERS, which was a precepting model developed by two members of the research team (D.H.R. and C.R.W.) and a faculty colleague and was designed to incorporate the Paul and Elder Critical Thinking Framework into the process of patient care [18,19]. The model entails eliciting the learner’s clinical reasoning process, facilitating reflection of the learner’s reasoning, delivering feedback, asking the learner to identify a related learning goal, and then providing the learner with support. These methods are similar in that both aim to improve a learner’s critical thinking skills. Pause, Predict, Ponder helps promote learner knowledge and retention, while LEARNERS is designed to further develop learner clinical reasoning skills. The process required within each method varies from one another in terms of level of preceptor involvement, order of steps, and type of interactions elicited.

Before the start of studying, each participant was given a pretest as a baseline assessment to assess, knowledge, self-efficacy, and perception of behavior related to the development topics. Participants were randomized into two groups and attended programming to teach them how to use each of the teaching models. Participants learned one method either via a traditional format (i.e., video recorded PowerPoint^®^ presentation) or microlearning and then learned the second method via the alternate format immediately after completing the first. In both groups, the traditional method was utilized first to teach the participants about Pause, Predict, Ponder or LEARNERS for groups 1 and 2, respectively. Both groups then crossed over to learn the other topic, this time using microlearning as the method. The meeting and assessments were held over a one-hour period held via Zoom (https://zoom.us , accessed on 20 November 2021) and moderated by one of two study investigators. Moderators were educated on the study protocol, preceptor development topics, and modalities to ensure standardization across all participants.

The traditional method consisted of a 30 min video-recorded PowerPoint^®^ of the topic. The 15 min microlearning session consisted of an interactive eLearning module created through the application “edApp” (www.edapp.com, accessed on 21 November 2021), a video example of a preceptor utilizing the precepting method with a student, and an infographic summarizing the method. The content and learning objectives within each learning method were the same regardless of the modality used. Participants were given the same assessment immediately after completion of the session and then again one month later. Satisfaction was also assessed during the immediate post-test.

Satisfaction scores were measured using a 4-point Likert scale to assess efficiency and effectiveness by asking preceptors to rate their experiences with each modality. The scales ranged from very ineffective (1) to very effective (4) or very inefficient (1) to very efficient (4), respectively. Knowledge change was assessed using ten multiple-choice questions created by the team members that developed the program content to assess each topic. Self-efficacy was assessed on a numerical scale ranging from zero to one hundred percent correlating to their level of confidence in performing skills related to the preceptor development topics. Preceptors were asked to rate their level of confidence to help facilitate learner clinical reasoning skills (LEARNERS) and knowledge and retention (Pause, Predict, Ponder) for both the traditional method and microlearning. Perceptions of behavior were self-reported and assessed how often preceptors reported precepting behaviors related to increasing learner knowledge and retention and clinical reasoning skills with a score of zero indicating the least frequent amount (never) and a score of three (daily) indicating the most frequent amount.

During the immediate post-assessment, participants were given the opportunity to supplement their satisfaction answers by providing short answer responses. Inductive coding and thematic analysis were utilized to assess these data [20,21]. A codebook was created by one team member, and a second team member applied the codebook to the data and added any new codes that emerged from the data. Inter-rater reliability (ICR) was determined at the question level to ensure consistent coding with the two coders [22]. The average ICR was 83.8% and all discrepancies were resolved among the coders. Pattern coding was then used to identify patterns in codes by grouping together codes with similar major themes [20,21].

Primary outcomes were satisfaction scores and change in knowledge, self-efficacy, and perceived behavior. Data were collected from Qualtrics (Provo, UT, USA) and analyzed using R [23]. The overall knowledge scores were compared using one-way repeated measures ANOVA and the Wilcoxon paired t-test. Descriptive statistics were utilized to summarize demographics, satisfaction data, and perception of behavior. Self-efficacy ratings were also assessed using one-way repeated measures ANOVA and Wilcoxon rank sum tests. Differences in satisfaction and perception of behavior between modalities were measured using a paired Wilcoxon t-test. Significance was set at *p*-value < 0.05.

## 3. Results

Twenty-five preceptors agreed to be enrolled in the study. All preceptors (100%) completed both the pre- and immediate post-questionnaires, and 23 (92%) completed the one-month post-questionnaires. No preceptors were excluded. Baseline characteristics are reported in Table 1.

### 3.1. Satisfaction

**Figure 1:**
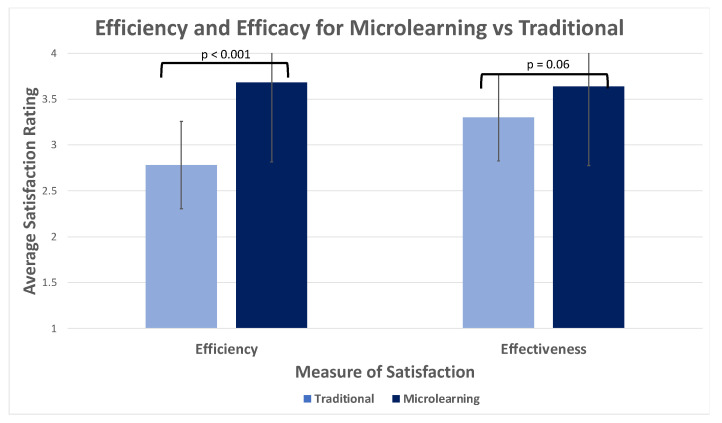
Efficiency and effectiveness of microlearning compared to the traditional method.

A comparison of medication satisfaction scores is represented in Figure 1. There was a statistically significant difference between the perceptions of efficiency, favoring microlearning (*p* < 0.001). There was no significant difference between modalities for the perception of effectiveness (*p* = 0.06). Overall, 72% of participants preferred microlearning compared to 20% who preferred the traditional method (*p* = 0.007). Forty-six percent of participants shared that microlearning helped the motivation to learn more as compared to 8% of those who stated that traditional learning helped more with this (*p* = 0.0023). Seventy-six percent reported feeling more engaged while using microlearning as compared to four percent when using the traditional method (*p* = 0.0033).

The most prominent theme that emerged during the inductive coding of modality preferences was that microlearning was more engaging than the traditional method. One participant commented, “I am a big fan of active learning, so I find using the microlearning methods more effective than PowerPoints.” Efficiency was the second most prevalent theme with another individual stating, “With a busy clinic schedule, short micro-learnings allow for me to learn during my lunch break and still allow time to catch up from my morning”.

Apart from suggesting no changes, the most common theme for changes to microlearning was the desire for interactive practice. One participant mentioned, “being able to practice myself and make it more interactive”. The most common theme for the traditional method was observation of interaction; for example, “It would have been nice to have a video role-play of the case/scenario instead of a slide with it all written out. The role-play is more effective”.

### 3.2. Knowledge and Self-Efficacy

There was a significant increase in overall knowledge scores pre- and immediate post and pre- and one-month post for both modalities (Table 2). When comparing knowledge outcomes between the microlearning and traditional method, there was no significant difference in scores in any of the three assessments. There was a significant increase in self-efficacy scores when comparing pre and immediate post for both modalities (Table 3). When comparing self-efficacy between the two different modalities, there were no significant differences seen when comparing average pre and immediate post scores for each learning topic.

### 3.3. Behavior

There was no significant difference in the frequency of self-reported precepting behaviors related to the topics pre and one-month post for reach modality, except in those who had learned the Pause, Predict, Ponder through the traditional method when asked how often they used strategies to increase learner retention of knowledge and comprehension (*p* = 0.046). The pre-assessment had an average score of 1.73 while the one-month post-assessment had an average score of 2.14. These significant findings comparing pre and one-month scores were not seen for those who had learned Pause, Predict, Ponder through microlearning (*p* = 0.299). When comparing microlearning and the traditional method directly for the one-month assessment, there was no significant difference for either the Pause, Predict, Ponder method or LEARNERS for any of the statements used to assess behavior. There was also no significant difference when comparing microlearning to the traditional method directly for both topics during the one-month post-assessment. This was true when evaluating how often preceptors reflected on learner clinical reasoning skills (*p* = 0.897) and how often they reported using clinical strategies to increase learner retention and comprehension (*p* = 0.306), which both assessed the LEARNERS topic. A similar finding comparing microlearning and the traditional method was seen when evaluating how often preceptors reflected on learner retention of knowledge and comprehension (*p* = 0.877) and the strategies they used to increase learner retention of knowledge and comprehension (*p* = 0.215), which both evaluated Pause, Predict, Ponder.

## 4. Discussion

This study evaluated the use of microlearning in preceptor development by assessing the satisfaction, knowledge, self-efficacy, and self-perceived behavior of pharmacy preceptors using two different learning topics in order to evaluate the effectiveness of microlearning. Prior studies have assessed microlearning in different health care disciplines almost exclusively with student learners. This study demonstrated increased preceptor satisfaction with microlearning over the traditional method. Additionally, both methods resulted in significant increases in knowledge and self-efficacy scores when comparing pre to post-assessment scores.

The overall satisfaction data are in support of using microlearning for preceptor development over a traditional video recorded PowerPoint^®^, with the majority of preceptors noting that they would prefer to use microlearning over the traditional method. These findings were also supported by the qualitative responses, wherein preceptors identified microlearning as a more engaging, effective, and efficient method to learn. High satisfaction with microlearning for faculty development has also been reported [15,16]. Satisfaction, while the lowest level of outcome on Kirkpatrick’s training evaluation model, informs quality improvement and is an important driver for preceptor engagement with learning materials given time barriers [7,24]. Preceptors indicated that active learning strategies and interaction used in microlearning increased attentiveness over passive participation. This suggests that the active microlearning strategies themselves may have helped preceptors to stay focused on the material they were learning. Higher education students on average maintain the greatest attention for 10–15 min during a 50-min lecture which suggests that microlearning may be a suitable learning method for engaging learners [25]. This idea is supported by our finding that preceptors report microlearning to be more engaging compared to the traditional method. Short answer feedback, however, also revealed perceived deficits in the traditional method. Preceptors were able to observe a preceptor interaction as a part of the microlearning session; however, this was not the case for the traditional method. This limitation should be acknowledged in the study design when considering future studies assessing the value of microlearning in preceptor development.

There were also comments with common themes that were found in both modalities such as the desire for live interactive practice which was excluded in both modalities due to limitations within the study. This finding suggests that simulated practice of skills learned in preceptor development programs should be considered and is in alignment with findings from a systematic review by Steinert and colleagues [26]. This review supports the idea that effective faculty development initiatives identify the application of knowledge and practicing skills learned as key features of effective faculty development programs [26]. One method to accomplish this through microlearning is to offer foundational knowledge through microlearning and follow-up with live application. The most common theme for the question regarding what preceptors would change about their learning modality for the traditional method was the observation of interaction. Preceptors were able to observe a preceptor interaction as a part of the microlearning session; however, this was not the case for the traditional method. The traditional method was entirely a 30-min voiceover PowerPoint^®^, so its structure as defined and predetermined by this study did not allow for the flexibility to add this component to the learning intervention. Altogether these findings suggest that the simulated practice of learned material and the diversity of educational methods within programs are key features of effective faculty development, which are in alignment with the findings from Steinert and colleagues on effective faculty development [26].

Although this study did not demonstrate a clear benefit of microlearning over a traditional method when evaluating knowledge, self-efficacy, or perception of behavior, it is important to confirm these findings in a larger number or population of preceptors and in other locations and schools of pharmacy in the United States. In addition to learning, self-efficacy, and behavioral outcomes, the length of programming is a relevant factor that must be taken into consideration when evaluating the effectiveness of microlearning. Qualitative and quantitative data from this study support microlearning as a more efficient learning method. A small minority of published microlearning studies assessed efficiency and effectiveness as an endpoint when measuring overall satisfaction, none of which included preceptor development [9]. A study by Narula et al. among clinical clerks found that 91% of students in the five-minute video clip microlearning group agreed that it was a time-effective way to learn about symptoms and diseases [27]. Similar to our findings, the majority of students (97%) in the experimental group agreed that it was also an effective way to learn as well [27]. Efficiency is convenient for educating those who access the material during time-sensitive situations or for busy clinicians [27]. Pharmacists may have limited time to access training due to balancing administrative, teaching, and patient care roles [1,2,3,4,5]. By shortening the time needed to learn new material, pharmacists can have additional time to work on other essential jobs and tasks that are necessary in their role. Shortened delivery time and increased satisfaction suggest that microlearning may be a preferred modality for preceptors over traditional methods. Additional study is needed to confirm these findings.

An increase in knowledge scores was noted after the use of microlearning and the traditional method in both the immediate post and one-month post-test. While there were positive changes over time for both modalities, there were no significant differences between conditions when directly compared to one another. The majority of the current literature supports the idea that the microlearning intervention results in increased knowledge over the respective control variables, whereas our data suggest that there was no difference [11]. It is also important to note that most of this literature examines student populations as opposed to preceptors, which was the population sampled in this study. The discrepancy in outcomes may be due to the variation in the types of microlearning used or the time allotted during the washout period between interventions and microlearning groups in each respective study, as differences in these variables between studies may have had an effect on learning and therefore knowledge scores. Preceptors were excluded if they had baseline knowledge of the topics. Further study is needed to determine whether having baseline learning prior to engaging in microlearning would result in a difference in knowledge change compared to traditional programming. At present, there are no comparative studies assessing knowledge change after microlearning in preceptor development. In medical students, Cheng et al. observed higher splint assessment raw scores after medical students watched a three-minute just-in-time training (JITT) video and noted a higher rate of successful splint application in comparison with reading in a medical textbook [9]. It is important to consider the variation between studies exemplified by the shorter duration of the microlearning intervention, the population being medical students, and their control group reading in a textbook instead of watching a voiceover PowerPoint^®^ [9].

Swartzwelder observed findings similar to those seen in our study in that there was no significant difference in nursing students’ overall course grades between the control and microlearning groups [28]. No change in the overall percentage was seen despite students in the study undergoing microlearning intervention receiving weekly questions via text compared to the traditional email message standard in the course [28]. It is important to consider that there may have been external factors outside of the microlearning intervention that influenced this overall outcome such as other assignments turned in for a grade throughout the semester.

Some microlearning studies have allowed for the revisitation of material by learners after the first review, which may prove to be beneficial for learners when attempting to commit material or information into their long-term memory. Evans evaluated student satisfaction and performance on exams after viewing screencasts an unlimited number of times to supplement in-class lecture material [29]. The implementation of the screencast into learning improved students’ subsequent exam scores compared to their previous scores [27]. Retrieval processes involved with distributed study may help with consolidating new material into long-term memory [30,31]. Allowing preceptors to revisit smaller blocks of material may result in better learning outcomes and should be assessed in future studies [30,32].

The increase in knowledge pre-post in the present study supports either microlearning or traditional methods for preceptor development. The decision between methods can be influenced by other factors impacting the overall learning experience or time and resources available. Under the circumstance of preceptor time limitations, it may be appropriate to consider utilizing microlearning as it may be a more efficient use of preceptor time. Additional studies in other and larger populations are needed to confirm the study findings.

While self-efficacy scores increased pre-post using both methods, there were no significant differences between methods. As with knowledge, it appears there are currently no comparable studies evaluating self-efficacy changes after microlearning for preceptor development. Ball and colleagues reported that students’ self-efficacy when identifying potential violence significantly improved after watching a video podcast on violent patient management during their emergency medicine clerkships compared to no intervention [33]. No overall significant differences in levels of self-efficacy in the present study suggest that either microlearning or the traditional method are acceptable methods to use for preceptor development. Additional inquiry in larger populations with repeat exposure to the content is needed to confirm these findings.

The frequency of perception of use of precepting behaviors related to the topics was similar between microlearning and the traditional method and nonsignificant for the majority of the measurements. It is unclear if a longer period prior to assessing behavior change or repetition of content would have resulted in a different outcome. There is a dearth of literature evaluating behavior change after microlearning for preceptor development. Richardson and colleagues sought to assess the behavioral changes of first-year nursing students through self-reporting [34]. Students chose to receive either a text message or an email regarding key class concepts in a first-year anatomy and physiology course. Students who had opted for short text messages reported it helped them to more frequently initiate their studies [34]. The behavior assessed in these students is different than the behavior that is necessary for assessment in health care professionals and preceptors. The significant finding for behavior in the statement measuring frequency for use of strategies to use to increase learner knowledge retention and comprehension in the Pause, Predict, Ponder method may indicate that microlearning may have more benefit in straightforward topics compared to more complex ones. Since there are similar levels of perceived behavior overall between microlearning and the traditional method, it may be reasonable to consider either method acceptable for preceptor development. More studies are needed to assess the perception of behavior change and actual behavior change in preceptor development and determine best practices.

Strengths of the study include the assessment of higher levels of training evaluation and the use of a randomized cross-over design, which was implemented to limit subject variability [18]. The inclusion of two different learning methods was utilized as a part of the randomized cross-over design, which was implemented to limit subject variability and control for different levels of complexity within different learning materials. There were some limitations of the study. The study was conducted across one school of pharmacy with a small sample size, limiting generalizability; however, preceptor training needs are often similar across institutions. Further study in larger populations of preceptors and preceptors from other schools and colleges of pharmacy is suggested. There is also the potential for bias that may impact the interpretation of results. Observer bias may play a role in overestimating or underestimating the preference for a particular learning method with subjective data results from the inductive coding and thematic analysis used to interpret qualitative data. Two coders were utilized to control for this potential bias. Convenience sampling also could have introduced bias as preceptors who volunteered to participate may have been more interested in microlearning. Pause, Predict, Ponder and LEARNERS had differences in terms of the number of steps involved (Pause, Predict, Ponder had less), their focus (knowledge and retention with Pause, Predict, Ponder versus clinical reasoning with LEARNERS), and complexity; however, this was controlled by the cross-over design. Behavior was self-reported versus directly observed and should be a focus of future inquiry. The knowledge change questions were limited in number, making it more difficult to detect a difference. Additionally, the preceptors did not have the opportunity to revisit the content from the microlearning sessions, which may have helped with knowledge retention. Further, more study is needed to assess efficiency from the trainer perspective regarding the preparation of materials. Further evaluation is needed to assess knowledge change after material repetition, the real-world behavior of preceptors, and student knowledge change.

## 5. Conclusions

This study comparing the application of microlearning to a traditional method of learning commonly used in preceptor development found that preceptors preferred microlearning when given the choice between microlearning and a traditional method of learning. No differences in overall knowledge change, self-efficacy, and perception of behavior between the two modalities were found. Quantitative analysis indicated that both modalities still led to an overall expected increase in knowledge and self-efficacy scores. The increased satisfaction from the microlearning group, similar knowledge and self-efficacy outcomes as a traditional method, and reduced delivery time suggest that microlearning may be an attractive option to consider when training busy preceptors. Additional studies in larger populations of preceptors are warranted to further explore and understand the benefit of microlearning in preceptor development as well as the resources required to deliver it.

## Figures and Tables

**Table 1:** Baseline demographics and characteristics of preceptors.

Baseline Characteristics	PPP Traditional and LEARNERS Microlearning (N = 12)	LEARNERS Traditional and PPP Microlearning (N = 13)
Age, years (mean ± SD)	38.6 ± 7.97	35.8 ± 5.4
Completed Educational Experience (% of total)		
Bachelor’s Degree (BSPharm, BS, BSN, BA)	6 (50%)	5 (38.4%)
Master’s (MS, MSN, MEd)	0 (0%)	3 (23.1%)
Doctoral (PhD, EdD)	1 (8.3%)	2 (15.4%)
Professional (PharmD, DNP, MD, JD, DMD, DDS)	10 (83.3%)	9 (69.1%)
Post Graduate Year 1 Residency	5 (41.7%)	7 (53.8%)
Post Graduate Year 2 Residency	3 (25%)	3 (23.1%)
Board Certification	3 (25%)	8 (61.5%)
Fellowship	1 (8.3%)	0 (0%)
Years Served as a Preceptor N (%)		
1–5 years	4 (33.3%)	4 (30.8%)
6–10 years	3 (25%)	5 (38.4%)
11–15 years	3 (25%)	0 (0%)
16–20 years	1 (8.3%)	2 (15.4%)
>20 years	1 (8.3%)	2 (15.4%)
Students Precepted per Year N (%)		
1–5 students	6 (50%)	6 (46.2%)
6–10 students	4 (33.3%)	5 (38.5%)
11–15 students	1 (8.3%)	1 (7.7%)
16–20 students	0 (0%)	0 (0%)
>20 students	1 (8.3%)	1 (7.7%)
Residents Precepted per Year N (%)		
0 residents	2 (16.7%)	4 (30.8%)
1–5 residents	8 (66.7%)	7 (53.8%)
6–10 residents	2 (16.7%)	2 (15.4%)
Total Learners Precepted in Career (mean ± SD)	38.6 ± 24.1	44.3 ± 31.1
Types of Learners Precepted N (% of total)		
First-year students	3 (25%)	4 (30.8%)
Second-year students	7 (58.3%)	8 (61.5%)
Third-year students	6 (50%)	9 (69.1%)
Fourth-year students	12 (100%)	13 (100%)
Residents	9 (75%)	9 (69.1%)
Other health professional students outside of discipline	2 (16.7%)	1 (7.7%)
Types of Preceptor Development Programs Completed N (% of total)		
Preceptor development programs at national conferences	6 (50%)	6 (46.2%)
National preceptor training programs	1 (8.3%)	1 (7.7%)
School-based training and development programs	11 (91.7%)	11 (84.6%)
Preceptor development programs for resident preceptors at my organization	8 (66.7%)	7 (53.8%)
Types of Preceptor Development Resources Accessed Regularly N (% of total)		
Continuing education seminars/webinars/workshops	12 (100%)	11 (84.6%)
Preceptor development books	2 (16.7%)	1 (7.7%)
Newsletter/written resources	6 (50%)	3 (23.1%)
Professional organization resources	6 (50%)	3 (23.1%)
Preceptor development online resources	7 (58.3%)	6 (46.2%)
None	0 (0%)	1 (7.7%)

PPP = Pause, Predict, Ponder.

**Table 2:** Comparison of preceptor knowledge scores between pre, immediate post, and one-month post assessments and between different learning modalities.

Microlearning vs. Traditional Knowledge Scores Across Pre, Immediate Post, and One-Month Post Assessments						
Assessment	Overall Score %	Microlearning Average Score %		Traditional Average Score %		*p*-Value
Pre score	37.2%	33.6%		40.8%		0.222
Immediate Post Score	76.4%	75.2%		77.6%		0.480
One-Month Post Score	52.1%	51.2%		53%		0.860
**Pre vs. Immediate Post vs One-Month Post Knowledge Scores for Microlearning and Traditional Modalities**						
Assessments	*p*-Value					
	Overall		Microlearning		Traditional	
Pre vs Immediate Post	<0.001		<0.001		<0.001	
Pre vs One-Month Post	<0.001		<0.001		<0.001	
Immediate Post vs One-Month Post	<0.001		<0.001		<0.001	

**Table 3:** Comparison of average preceptor self-efficacy ratings on a zero to one hundred scale for self-efficacy statements across pre, immediate post, and one-month post assessments between microlearning and traditional learning modalities.

Self-Efficacy Statement	Topic	Self-Efficacy Score (%)			*p*-Value						Self-Efficacy Score (%)		*p*-Value
		Pre	Immediate Post	One-Month Post	Microlearning			Traditional			Immediate Post		
					Pre vs. Immediate Post	Pre vs. One-Month Post	Immediate Post vs. One-Month Post	Pre vs. Immediate Post	Pre vs. One-Month Post	Immediate Post vs. One-Month Post	Traditional	Microlearning	Microlearning vs. Traditional
Identify case presentation methods to increase learner clinical reasoning skills	LEARNERS	51.24	72.92	58.67	<0.001	0.008	0.182	<0.001	0.023	0.059	64.97	61.54	0.059
Use case presentation methods to increase learner clinical reasoning skills	LEARNERS	47.21	70.6	61.56	<0.001	<0.001	0.646	<0.001	<0.001	0.982	62.14	62.51	0.982
Identify strategies to increase learner retention of knowledge and comprehension	PPP	48.56	74.84	62.11	<0.001	<0.001	0.011	<0.001	<0.001	0.149	62.2	64.51	0.149
Use strategies to increase learner retention of knowledge and comprehension	PPP	50.92	75.52	59	<0.001	0.005	0.002	<0.001	<0.001	0.038	63.54	64.81	0.038

PPP = Pause, Predict, Ponder.

## Data Availability

The data presented in this study are available on request from the corresponding author. The data are not publicly available due to privacy reasons.
